# Pheochromocytoma Presenting as Acute Heart Failure Leading to Cardiogenic Shock and Multiorgan Failure

**DOI:** 10.1155/2011/596354

**Published:** 2011-05-10

**Authors:** Jochen Steppan, Julia Shields, Ralph Lebron

**Affiliations:** ^1^Department of Anesthesiology and Critical Care Medicine, The Johns Hopkins Medical Institutions, 600 North Wolfe Street, Baltimore, MD 21287, USA; ^2^The Baltimore VA Medical Center, University of Maryland Medical Center, Baltimore, MD 21201, USA

## Abstract

Pheochromocytoma is an endocrine tumor classically presenting with headache, paroxysmal hypertension, and palpitations. We discuss the case of a young male, presenting with acute heart failure and cardiogenic shock requiring stabilization with an intra-aortic balloon pump and a combination of ionotropes and vasopressors. Pheochromocytoma was diagnosed by CT scan, as well as urine and plasma metanephrines. After pretreatment with phenoxy-benzamine, the patient underwent adrenalectomy with subsequent cardiovascular stabilization and full recovery. Unfortunately, pheochromocytoma often remains undiagnosed. Given the ample diagnostic tools and good prognosis when treated suitably, the diagnosis should be entertained early in patients, presenting with unexplained cardiovascular compromise.

## 1. Introduction

Pheochromocytoma is an endocrine tumor typically located in the adrenal glands which leads to catecholamine release and profound multiorgan involvement [1]. Typical symptoms include headache, palpitations, excessive sweating, and intermittent or persistent hypertension. Other symptoms such as abdominal pain, shock, respiratory distress syndrome, pulmonary edema, hyperthermia, and cardiogenic shock occur less frequently [2–4]. The cardiovascular manifestations of pheochromocytoma can also include electrocardiographic changes, conduction disturbances, left ventricular hypertrophy, T-wave changes, and cardiac arrhythmias. Apart from pheochromocytoma, catecholamine excess may occur from stress, solvent abuse, overuse of adrenaline inhaler in patients with asthma, or prolonged amphetamine use [5].

## 2. Case Report

A 42-year-old Hispanic man presented to an outside hospital with a history of diffuse and intermittent abdominal discomfort, lasting minutes to hours. It was associated with nausea, vomiting, and abdominal fullness. On the day of presentation, he developed acute shortness of breath, diaphoresis, and epigastric discomfort.

In the Emergency Department at the outside hospital, the patient was found to be in acute respiratory distress with severe hypoxemia, requiring emergent intubation. An EKG revealed sinus tachycardia at 156 beats per minute, hyperacute T-waves in the anterior leads, and ST depressions inferiorly. Transthoracic echocardiography revealed an ejection fraction (EF) of less than 20% with normal left ventricular diastolic filling and normal valvular function. Right ventricular size and function were normal, and there was no pericardial effusion. Emergent left heart catheterization revealed normal coronary arteries, an EF of 15% with global hypokinesis, and a left ventricular end-diastolic pressure of 45 cm H_2_0. During catheterization, he became profoundly hypotensive (60 s/30 s) requiring a combination of pressors and ionotropes as well as an intra-aortic balloon pump (IABP) for additional cardiovascular support. Subsequently, he was transferred to our cardiac intensive care unit for further management.

Pertinent positives findings on transfer: intubated and sedated thin man, temperature 36.8°C, BP 110/90 mmHg, pulse 130 bpm, respirations 15/min, pulse oximetry 100% on a ventilator. Pulmonary exam showed bilateral coarse breath sounds with scattered rales. Cardiovascular exam revealed a tachycardic, regular rhythm, with no murmurs, rubs, or gallops, but jugular venous distention to the angle of the mandible. 

Laboratory studies revealed WBC 20,900/mcL, hemoglobin 19 g/dL, BUN 21 mg/dL, creatinine 2.45 mg/dL, glucose 74 mg/dL, AST 616U/L, ALT 653 U/L, alkaline phosphatase 185 U/L, total bilirubin 1.9 mg/dL, lactate 7 mmol/L, BNP 488 pg/mL, troponin I 69 ng/mL, urine toxicology negative, TSH 0.9 mIU/mL, free thyroxine 1.1 ng/mL, and HIV-1 and 2 antibody negative. 

**Figure 1:**
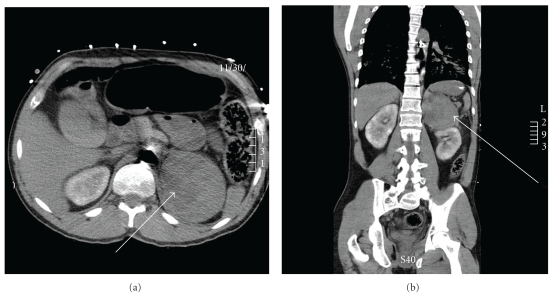
Noncontrast-enhanced abdominal CT scan showing the 9.2 × 7.6 cm soft tissue mass arising from the left adrenal gland (white arrow). (a) Transverse image, (b) Axial image.

The patient was admitted to the cardiac intensive care unit, and we continued cardiovascular support with a combination of pressors and ionotropes as well as the IABP. A chest film revealed bibasilar infiltrates and a normal cardiac silhouette. A transesophageal echo confirmed the EF of 15% with normal left ventricular diastolic filling and normal valvular function and no signs of Takatsubo cardiomyopathy present. As the patient remained minimally responsive to therapy, a CT scan was ordered to exclude unrecognized bleeding or an occult source of infection. The abdominal CT scan without contrast revealed a 9.2 × 7.6 cm soft tissue mass arising from the left adrenal gland (Figure 1). 

**Figure 2:**
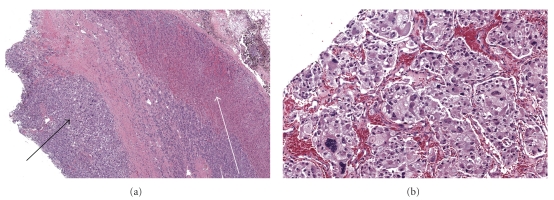
Microscopic images showing pathologic slides of the excised pheochromocytoma (a) Pheochromocytoma predominantly consisting of pleomorphic chief cells with round, hyperchromatic nuclei (black arrow) adjacent to normal adrenal tissue (white arrow). (b) Higher magnification showing zellballen (nests) surrounded by a fibrovascular stroma, with the cells showing a typical “salt and pepper” chromatin and abundant granular amphophilic cytoplasm.

Catecholamines were drawn, showing very highly elevated levels of epinephrine (24,186 pg/mL, normal <84 pg/mL) and norepinephrine (24,691 pg/mL, normal <420 pg/mL) with comparably less elevated levels of dopamine (848 pg/mL, normal <60 pg/mL). Furthermore, urine and plasma metanephrines were elevated 250-fold and 500-fold over their respective normal ranges. Given these laboratory and imaging findings, pheochromocytoma was considered the most likely diagnosis. The patient was pretreated with phenoxybenzamine for three days and underwent uncomplicated adrenalectomy on hospital day seven. After resection and pathological confirmation of a pheochromocytoma (Figure 2), the patient's cardiovascular status rapidly stabilized. Follow-up pan-CT did not reveal any metastatic disease. Pressors and ionotropes were discontinued, the IABP was removed, and the patient was successfully extubated. A repeat echocardiogram on day twelve showed an EF of 45–50%. The patient was discharged on day 13 after near full recovery to be followed up for genetic testing of Germ line mutations, which came back negative.

## 3. Discussion

Pheochromocytoma is a great disease imitator. The classic triad of headache, palpitations, and paroxysmal hypertension is not always present. Recognition requires a high index of suspicion as it may present atypically including dilated cardiomyopathy, pulmonary edema, sudden death, severe sepsis, myocarditis, acute myocardial infarction, or, as in this case, cardiogenic shock. This may at least partially explain why is still a relatively uncommon diagnosis, despite being present in up to 1 : 2,000 autopsies, as shown by a retrospective study of 38,596 autopsies from Australia by McNeil et al. [6]. In another retrospective study from the Mayo Clinic, from the years from 1950 through 1979, 11 cases of pheochromocytoma were diagnosed in an average population of 45,800 [7]. This represents an annual incidence rate of 0.8 nonfamilial pheochromocytomas per 100,000 person-years. Of those 11 cases, 5 were diagnosed initially at autopsy [7]. These results are similar to the ones obtained by the Swedish cancer registry, which found that 42% of pheochromocytomas were first diagnosed after death [8]. Weather all of the tumors discovered at autopsy are active and clinically relevant remains unclear. 

The pathogenesis of catecholamine-induced cardiomyopathy resulting in cardiogenic shock as seen with advanced pheochromocytoma is multifactorial. Myocardial dysfunction due to catecholamine-related injury of myocardial fibers appears to be the most common pathomechanism. Catecholamines can cause tachycardia-induced cardiomyopathy or may aggravate existing cardiac conditions [9]. Furthermore, excessive adrenergic stimulation may cause coronary vasoconstriction and vasospasm, resulting in myocardial ischemia and subsequent cardiomyopathy [10]. 

Catecholamines exert a receptor-mediated effect on the myocardium. Long-term elevation of catecholamine levels lead to downregulation of beta-adrenergic receptors, thereby inducing suboptimal functioning of myofibers as well as a decreased number of contracting units [5]. Furthermore, oxidized catecholamines can increase sarcolemmal membrane permeability, leading to calcium influx and give rise to profound changes in intracellular calcium mechanisms [11]. This can consequently result in an acute myocarditis, with diffuse interstitial inflammatory infiltrates and myocardial necrosis as well as decreased cardiac output. In the presence of hypovolemia, decreased cardiac output can culminate in cardiogenic shock [11], as seen in this patient.

Early diagnosis remains difficult in patients with atypical presentations such as abdominal pain, respiratory distress, pulmonary edema, hyperthermia, myocarditis, myocardial infarction, or cardiogenic shock. In these patients, a high index of suspicion is vital to timely diagnosis. In this case, the patient's history of intermittent abdominal pain led to an early CT scan and discovery of the adrenal mass. Subsequent acquisition of the highly elevated plasma catecholamines, as well as urine and plasma metanephrines, reinforced the working diagnosis of pheochromocytoma. Histology of the tumor verified the diagnoses by showing the classical pattern of pleomorphic cells with round hyperchromatic nuclei and granular cytoplasm that are forming nests (zellballen) surrounded by a fibrovascular stroma. Malignant disease, which can only be determined by metastatic spread of the tumor was not present in our patient.

Treatment of pheochromocytoma initially focuses on hemodynamic stabilization of the patient with aggressive intravascular fluid resuscitation as first line therapy, complemented by vasopressors and ionotropes as well as an IABP for additional cardiovascular support if needed. This is then followed by medical treatment with an alpha-blocker such as phenoxybenzamine and a beta-blocker such as esmolol. The alpha blocker should be started at a low dose and uptitrated before initiation of beta-blocker therapy to avoid unopposed alpha receptor stimulation by the excreted catecholamines. This medical therapy reduces the risk of intraoperative hemodynamic changes during tumor resection [12, 13]. Furthermore, patients with sporadic pheochromocytoma should be advised to undergo genetic testing for germ line mutations to identify pheochromocytoma-associated syndromes [14]. 

Unfortunately, pheochromocytoma remains undiagnosed in many patients presenting with atypical symptoms despite adequate diagnostic tools, potentially leading to lethal outcomes. Given the ample treatment options that greatly improve survival, it is essential to sustain a high index of suspicion to entertain the diagnosis early in patients with atypical presentations such as unexplained acute heart failure to improve the outcome for the individual patient.

## 4. Conclusion

Pheochromocytoma is a great disease imitator, and it remains undiagnosed in many patients presenting with atypical symptoms—despite being present in up to 1 : 2,000 autopsies [6]. Recognition requires a high index of suspicion as it may present atypically including dilated cardiomyopathy, pulmonary edema, sudden death, severe sepsis, myocarditis, acute myocardial infarction, or, as in this case, cardiogenic shock. Early diagnosis is essential to initiate adequate therapy and reverse cardiomyopathy at an early stage.

##  Funding 

This research did not receive any specific grant from any funding agency in the public, commercial, or not-for-profit sector.

##  Conflict of Interests 

There is no conflict of interest that could be perceived as prejudicing the impartiality of the research reported.
